# Recent Advances in Wearable Potentiometric pH Sensors

**DOI:** 10.3390/membranes12050504

**Published:** 2022-05-09

**Authors:** Yitian Tang, Lijie Zhong, Wei Wang, Ying He, Tingting Han, Longbin Xu, Xiaocheng Mo, Zhenbang Liu, Yingming Ma, Yu Bao, Shiyu Gan, Li Niu

**Affiliations:** 1 School of Civil Engineering, c/o Guangzhou Key Laboratory of Sensing Materials & Devices, Center for Advanced Analytical Science, School of Chemistry and Chemical Engineering, Guangzhou University, Guangzhou 510006, China; yitiantang@e.gzhu.edu.cn (Y.T.); wangw@gzhu.edu.cn (W.W.); ccyhe@gzhu.edu.cn (Y.H.); tinghan@gzhu.edu.cn (T.H.); longbinx@gzhu.edu.cn (L.X.); gdmoxiaocheng@e.gzhu.edu.cn (X.M.); cczbliu@gzhu.edu.cn (Z.L.); ccymma@gzhu.edu.cn (Y.M.); baoyu@gzhu.edu.cn (Y.B.); ccsygan@gzhu.edu.cn (S.G.); 2 School of Computer Science and Cyber Engineering, Guangzhou University, Guangzhou 510006, China

**Keywords:** ion-selective electrode, pH sensors, wearable sensors, potentiometric sensors

## Abstract

Wearable sensors reflect the real–time physiological information and health status of individuals by continuously monitoring biochemical markers in biological fluids, including sweat, tears and saliva, and are a key technology to realize portable personalized medicine. Flexible electrochemical pH sensors can play a significant role in health since the pH level affects most biochemical reactions in the human body. pH indicators can be used for the diagnosis and treatment of diseases as well as the monitoring of biological processes. The performances and applications of wearable pH sensors depend significantly on the properties of the pH–sensitive materials used. At present, existing pH–sensitive materials are mainly based on polyaniline (PANI), hydrogen ionophores (HIs) and metal oxides (MO_x_). In this review, we will discuss the recent progress in wearable pH sensors based on these sensitive materials. Finally, a viewpoint for state–of–the–art wearable pH sensors and a discussion of their existing challenges are presented.

## 1. Introduction

Wearable sensor devices have received extensive attention since they enable the continuous and close monitoring of an individual’s situation without interrupting or limiting the user’s movements [1,2,3,4,5,6,7,8,9,10]. Real-time information that reflects an individual’s physiology and health status can be obtained through the continuous monitoring of biochemical markers in biological fluids, such as sweat, tears and saliva [11,12,13,14,15,16,17]. In addition, wearable sensors play a vital role in enabling personalized medicine by continuously collecting human data and capturing meaningful changes in health status in a timely fashion for preventive interventions in situations that may arise [2,18,19,20].

At present, the reported entities that can be detected by wearable bio(chemical)sensors involve multiple species, including pH, Na^+^, K^+^, Ca^2+^, NH_4_^+^, Cl^−^, glucose, dopamine, urea, etc. [1,5,21,22,23,24,25,26,27,28,29,30,31]. Among them, the detection of pH is particularly significant—it is used as a routine indicator because it participates in most biochemical reactions in the human body [32,33,34]. Moreover, monitoring pH in the body can help manage chronic wounds and prevent infection processes [2,35,36]. The glass electrode has traditionally been the most widely used pH sensor. However, glass electrodes have some inherent disadvantages, such as high temperature instability, acid-base error, high impedance and difficulty in miniaturization, which limits their further application [37,38]. The latter challenge in integration hiders the application of traditional liquid–junction pH electrodes in wearable devices. In general, focused electrochemical wearable sensors require flexible devices [19,39,40,41].

In recent years, many new solid–contact pH sensors have been developed to replace glass pH electrodes for miniaturization and integration. These new materials concentrate on organic polymers [42,43,44,45], hydrogen ionophores [28,46], carbon nanotubes [47] and metal oxides [48,49,50,51,52], which have proven to be promising pH-sensitive materials. In this review, we will discuss the recent advances of these pH-sensitive materials in wearable sensors with an emphasis on the response mechanism and analytical performances. Finally, the existing challenges and an outlook for the wearable pH sensors will also be discussed.

## 2. The Influence of pH on Human Health

Testing the pH of human biofluids fluids can reveal a great deal of health–related biological information. An abnormal sweat pH reflects skin lesions, such as irritant contact dermatitis, acne vulgaris, atopic dermatitis, etc. [53,54,55]. Many cellular processes and enzymatic reactions are also dependent on pH. For example, in one study, it was found that living cells were destroyed and the formation of tumors was potentially promoted when the body fluid pH dropped from 6.9 to 6.5 [33]. In addition, an acidic environment has been shown to inflame blood cells and reduce oxygen levels, which impairs cells’ metabolic state and hinders the function of DNA and respiratory enzymes, leading to kidney, liver and sweat gland failure [56,57,58,59]. For acute wounds in the body, weak alkalinity has been shown to be more conducive to the growth of bacteria [60,61,62]. Therefore, the management of chronic wounds and the prevention of infection processes can be achieved by monitoring the pH value of the wounds [61,62,63,64,65]. Moreover, the pH levels of body fluids, such as sweat, tears, urine and saliva, can also be used as a basis for the early diagnosis of disease. For example, the sweat from cystic fibrosis patients is alkaline with a pH up to ~9 [32]. In conclusion, the pH value of the human body is closely related to health. Therefore, the use of wearable pH sensors can provide necessary information for the early detection of many diseases.

## 3. pH-Sensitive Materials and Wearable Sensors

### 3.1. Polyaniline (PANI)

PANI is one of the most widely used pH–sensitive materials, owing to its strong pH sensitivity [43], ionic and electron mixed conductivity [66,67], chemical stability and low cost [42]. PANI is often prepared by the direct oxidation of aniline through chemical oxidants or by electrochemical polymerization on electrode substrates [33,42,45,68]. Direct electro–polymerization on various electrode surfaces is beneficial for the preparation of miniaturized sensing chips.

**Figure 1:**
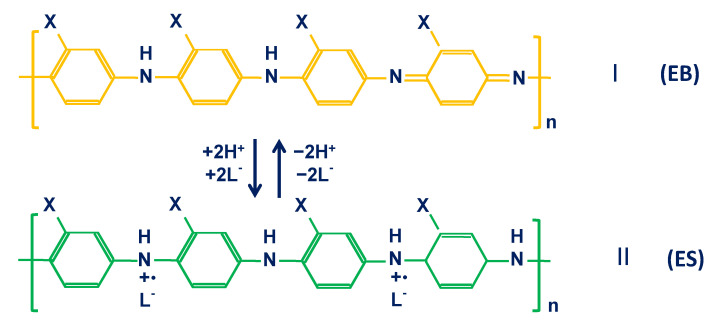
The mechanism of pH response for PANI and PANI derivates (X = H, CH_3_, C_2_H_5_ or C_3_H_7_) illustrating the protonation and deprotonation of the half–oxidized emeraldine–base (EB) and half-oxidized emeraldine salt (ES) forms. Reprinted with permission from [43], Copyright (2002) Elsevier.

pH–response mechanism for PANI. The pH response of PANI can be considered as a reversible protonation and deprotonation process. It is well known that PANI has three basic oxidation states: fully reduced leucoemeraldine base (LEB), half–oxidized emeraldine base (EB) and fully oxidized pernigraniline base (PNB) [43]. Among them, EB can be protonated to form conductive emeraldine salt (ES) due to the presence of amine and imine groups. The reversible protonation and deprotonation of EB and ES confer pH–sensitivity in PANI [43,54,69] (Figure 1). This reversible reaction is accompanied by a coupled transfer of protons and electrons, which is the origin of the Nernstian response for PANI–based potentiometric pH sensors [44,45].

**Figure 2:**
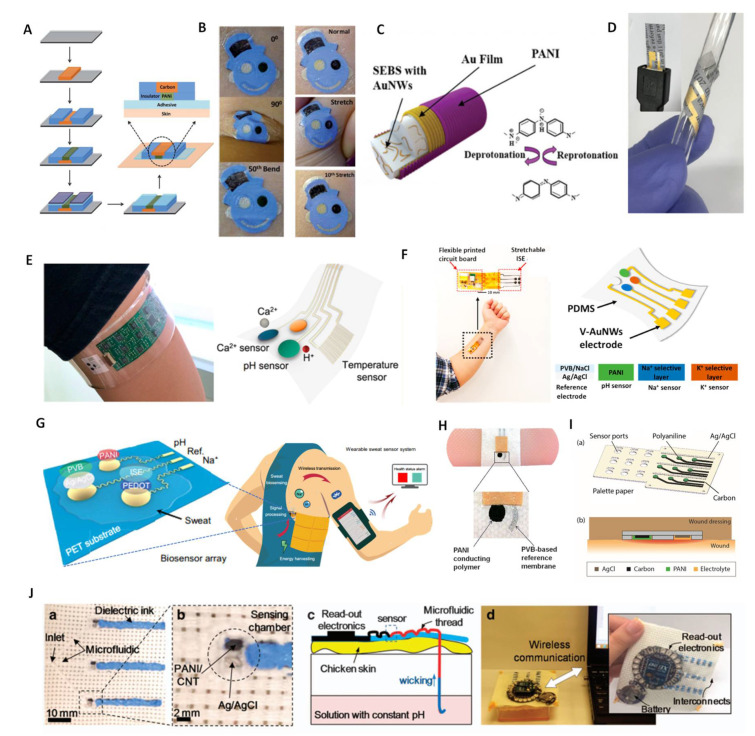
PANI–based wearable pH sensors. (**A**) The fabrication steps for PANI–based pH sensors on a tattoo substrate. (**B**) The tattoo applied to the cubital fossa at different bending and stretching states and cyclic tests. Reprinted with permission from [54], Copyright (2013) Royal Society of Chemistry. (**C**) The structure of a fiber pH sensor deposited by PANI. Reprinted with permission from [70], Copyright (2020) Royal Society of Chemistry. (**D**) Paper–based pH sensor under bending tests. Reprinted with permission from [71], Copyright (2017) Elsevier. (**E**) Wearable sensor array with PANI electrode integrated on PET substrate. Reprinted with permission from [72], Copyright (2016) American Chemistry Society. (**F**) Wearable sensor array with PANI electrode integrated on PDMS substrate. Reprinted with permission from [22], Copyright (2020) American Chemistry Society. (**G**) Self–powered wearable sensor with PANI electrode. Reprinted with permission from Science Advances [73], Copyright (2020) American Association for the Advancement of Science. (**H**) Bandage–based pH sensor with PANI electrode. Reprinted with permission from [74], Copyright (2014) John Wiley and Sons publications. (**I**) pH sensor array on paper substrate with self–aligned encapsulation. Reprinted with permission from [69], Copyright (2016) Elsevier. (**J**) A multiplexed microfluidic thread–based pH sensor: (**a**,**b**) structure of thread-based sensor; (**c**) cross–sectional model for in vitro pH measurement on the skin; (**d**) an integrated sensing system utilizing a wireless communication system. Reprinted with permission from [75], Copyright (2016) Spring Nature.

PANI–based wearable pH sensors. PANI has been widely used in wearable devices due to its easy deposition and high flexibility. Here, we will review the cases where PANI was used for wearable pH sensors. In 2013, Wang et al. reported on a tattoo–based pH sensor that was applied by attaching PANI to human skin through screen-printing technology [54]. PANI film was obtained by electro–polymerization with 25 cycles in aniline/HCl solution. The entire fabrication process is shown in Figure 2A. The authors examined the potential response of the tattoo sensor after extreme deformation by the cubital fossa (Figure 2B). The sensitivity of prepared tattoo sensor was significantly increased from −52.8 to −57.5 mV/pH at pH a range of 3–7 after bending 50 times and stretching 40 times, respectively. This enhanced sensitivity resulted from the expansion and reorientation of the crystalline and amorphous phases of PANI. The tattoo sensor had excellent flexibility and was able to meet the needs of various human movements; it was successfully applied for testing the pH value of sweat on neck, wrist and lower back. Recently, Cheng and coworkers developed a highly stretchable nanofiber pH sensor, which was prepared by the electrodeposition of PANI on gold fibers [70] (Figure 2C). The prepared fiber–based pH sensors featured a high stretchability and maintained normal operation under 100% strain with a response slope of −60.6 mV /pH in a pH range of 4–8. Lee et al. proposed a flexible paper–based PANI pH sensor that exhibited ultra–flexible and biodegradable characteristics [71] (Figure 2D). This paper–based pH sensor had a high sensitivity (−58 mV/pH in the pH range of 2–12) with a fast response time (<10 s) and excellent selectivity. The works described above demonstrated the excellent flexibility and tensile properties of PANI, which can be deposited on various substrates to prepare different flexible sensors with considerable application prospects in the wearable field.

In 2016, Javey and coworkers reported on a fully integrated multiplex sensor for the simultaneous detection of Ca^2+^ and pH in sweat [72] (Figure 2E). PANI was used as the pH–sensitive material. The wearable device constituted detection, data processing and transmission elements and was fabricated on a flexible PET substrate combined with electronic integration technology. The sensor had a sensitivity of −63.3 mV/pH with a relative standard deviation (RSD) of 2.3%. The wearable device was worn on the human body to achieve the non–invasive and continuous monitoring of sweat pH. The results were consistent with those of a commercial pH meter, indicating that the wearable device could be used for the early diagnosis of diseases. Subsequently, Cheng et al. reported on a sensor array on flexible PDMS in which the pH sensing element was electrically polymerized PANI film [22] (Figure 2F). The pH response potential did not change significantly as the sensing array was stretched by 30%, which again showed the good flexibility of PANI. The detection slope of the sensor in the pH range of 4–8 was −56.2 mV/pH. The flexible sensor array realized the detection of pH changes successfully during 30 min of exercises. Song and coworkers reported on a self–powered wearable sensing platform, including a PANI–based pH element, a printed circuit board–based freestanding triboelectric nanogenerator and an electronic integration chip [73] (Figure 2G). This self–powered system could provide the electric supply from human movement, ensuring that all sensing units worked for real–time monitoring. Physiological information, such as pH value, was obtained successfully in human tests. The work described above demonstrates the advantage of PANI in electronic integration for the large–scale fabrication of flexible sensing arrays.

In addition to in vitro testing, PANI–based pH sensors have also been used to build personalized medical devices. Wang and coworkers proposed a wearable bandage for the pH monitoring of wounds in which the pH sensing component was a screen–printed PANI flexible electrode [74], as shown in Figure 2H. The pH bandage sensor displayed a Nernstian response (−58.5 mV/pH) with an RSD of only 1.2% over the physiologically relevant pH range (5.5–8.0). The sensor successfully tested the pH on the wound, which facilitates an avenue towards the realization of telemedicine. Subsequently, Ziaie et al. used commercial palette paper as a substrate to prepare low–cost PANI-based flexible pH sensors for the detection of chronic wounds [69] (Figure 2I). The prepared paper–based sensor demonstrated a sensitivity of −50 mV/pH over a pH range of 4–10 and maintained potential stability for 24 h, which was consistent with the change time for the wound dressing. Additionally, Mostafalu et al. extended wearable pH sensors to in vivo detection [75]. A mixture of PANI, carbon nanotubes (CNTs) and carbon nanopowders was injected into fibers to prepare thread–based electrodes. An optical image of the prepared multiplexed microfluidic pH sensors is shown in Figure 2J. The thread was assembled on chicken skin to mimic subcutaneous measurements (Figure 2J(c)). The test data were presented on an external computer via a wireless system (Figure 2J(d)). The thread-based electrode showed a rapid response in subcutaneous tests, with a sensitivity close to the Nernstian slope, and the sensor remained stable for up to 4 h. In addition, the electrode was implanted into the stomach of a mouse and the pH value of gastric juice was detected successfully, which demonstrated the feasibility of a thread-based pH sensor as an implantable device. The work described above emphasizes the potential of PANI as a wearable pH sensor in personal medicine.

In addition to PANI, polyurethane (PU) has also been used for the preparation of wearable pH sensors. Dahiya and coworkers reported on a stretchable pH sensor based on graphite–PU [53]. The prepared sensor had a sensitivity of −11.1 ± 5.8 mV/pH, maximum response time of 5 s and good selectivity. The pH sensor was robust, with stretching up to 53% strain and more than 500 cycles under 30% strain. Its flexibility and low cost allow this work to be extended to other biomarkers.

In summary, PANI as a pH–sensitive material has excellent flexibility, good integration ability, high sensitivity, a short response time and low cost; thus, it has achieved intensive applications in the wearable field. However, the biotoxicity of PANI sensing materials could be a risk for practical use. Although some reports have claimed the biocompatibility of PANI [69], possible impurities, such as low–molecular–weight byproducts (benzidine), in PANI could have significant carcinogenic effects [76]. Therefore, the rational design of the structure of the wearable sensor should be emphasized, for example, by adding a microfluidic diversion system for sweat and an insulating layer between the skin and the sensing materials. In addition, the biocompatibility of PANI also requires further validation, which is critical for in vivo testing.

### 3.2. Hydrogen Ionophores (HIs)

Hydrogen ionophore (HIs) are proton acceptors for H^+^ recognition. Many solid contact ion–selective electrodes (SC–ISEs) based on different HIs have been tested in vitro as well as in various real samples. Most of them show a wide pH detection range and excellent selectivity, which are comparable to or in some cases even better than glass electrodes [77,78]. Compared with the fragility and difficulty in the miniaturization of glass pH electrode, HIs exhibit the ability of integration and thus have obvious advantages in wearable sensors.

**Figure 3:**
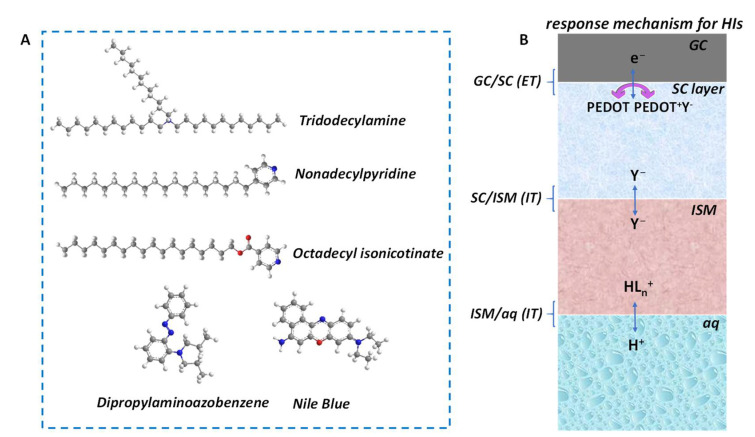
(**A**) Representative HIs include tridodecylamine, nonadecylpyridine, octadecyl isonicotinate, dipropylaminoazobenzene and Nile blue. (**B**) The pH–response mechanism of HI–based SC–ISEs. GC—glass carbon electrode substrate; SC—solid contact; ISM—ion-selective membrane; ET—electron transfer; IT—ion transfer; aq—aqueous solution.

***pH response mechanism for HIs***. The response mechanism of HIs to H^+^ can be described by classic ion–selective membrane (ISM)-based SC–ISEs. There are a few common His that are used for H^+^ recognition, including tridodecylamine (hydrogen ionophore I), 4–nonadecylpyridine, octadecyl isonicotinate, dipropylaminoazobenzene and Nile blue (Figure 3A). Taking tridodecylamine as an example, with typical poly(3,4–ethylenedioxythiophene) (PEDOT) as solid contact (SC) layer, the H^+^ response process can be described as follows [41]:

PEDOT^+^Y^−^ (SC) + H^+^ (aq) + e^−^ (GC) ⇋ PEDOT (SC) + Y^−^ (ISM) + H^+^ (ISM) (1)

where PEDOT^+^Y^−^ (SC) and PEDOT (SC) represent the oxidation and reduced states of PEDOT in the SC phase, respectively; H^+^ (aq) and H^+^ (ISM) are the concentrations in the measured aqueous solution and ISM phase, respectively; and Y^−^ is the doped anion. Equation (1) represents the proton response process (Figure 3B). As the target ions come into contact with the ISM, H^+^ can complex with the HIs in the ISM and pass through the membrane phase. Subsequently, the SC undergoes a redox reaction with PEDOT^+^Y^−^ doped with exchange anions, thereby converting the ionic signal into an electronic signal. The whole reaction involves the charge transfer balance of the three interfaces of GC/SC, SC/ISM and ISM/aq. $E_{SC}^{GC}$, $E_{ISM}^{SC}$ and $E_{aq}^{ISM}$ are the interface potentials of GC/SC, SC/ISM and ISM/aq, respectively. The sum of the three interface potentials is the total potential of the SC–ISEs, as shown in Equation (2). $E_{SC}^{GC}$ and $E_{ISM}^{SC}$ are constant values, since the components of SC and ISM are fixed such that the sum of the parameters other than the H^+^ concentrations are constant (*k*). Therefore, the total potential of SC–ISEs can be described as the Nernst equation related to H^+^ concentration, as follows:

(2) $$E=E_{SC}^{GC}+E_{ISM}^{SC}+E_{aq}^{ISM}=k+\frac{RT}{F}ln{\left[H^{+}\right]}_{aq}$$

where *R*, *T* and *F* represent the gas constant, temperature and Faradaic constant, respectively, and [H^+^]_aq_ is the concentration of H^+^ in the test aqueous solution.

**Figure 4:**
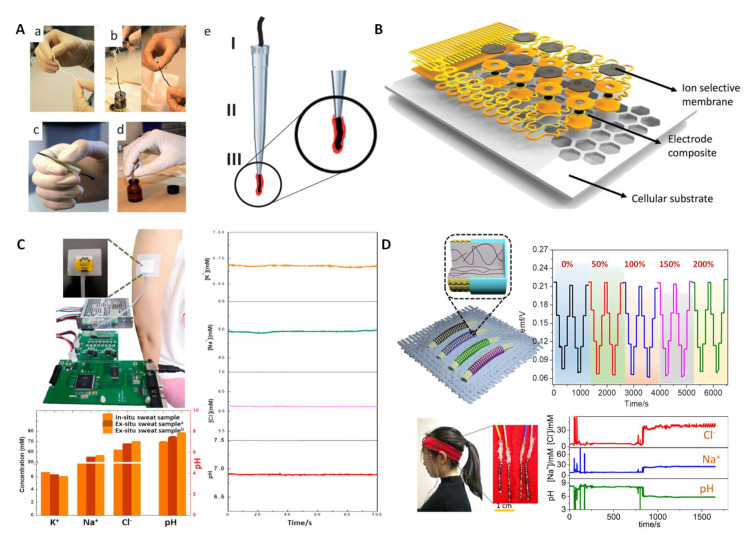
HI–based wearable pH sensors. (**A**) The steps for constructing CNT–cotton–based pH sensing electrodes. Reprinted with permission from [28], Copyright (2013) Royal Society of Chemistry. (**B**) Stretchable ion sensor array with an open cellular structure. Reprinted with permission from [46], Copyright (2017) John Wiley and Sons publications. (**C**) A paper–based multichannel SC–ISE for pH and other ion analysis in sweat. Reprinted with permission from [79], Copyright (2019) Elsevier. (**D**) Fiber–based wearable potentiometric sensor and its carry–over test from 0 to 200%. Reprinted with permission from [80], Copyright (2020) Royal Society of Chemistry.

HI–based wearable pH sensors. There are only a few reports on HI–based wearable pH sensors. In 2013, Andrade and coworkers proposed a simple and generalized approach for building wearable sensors using commercial cotton yarn [28]. The specific preparation process is shown in Figure 4A. Among them, H^+^ was recognized by the tridodecylamine ionophore. The CNT–cotton sensors were similar to those obtained with traditional SC–ISEs. Moreover, these sensors could be immobilized on a band–aid, indicating that this approach could be easily implemented in a wearable device. Rogers et al. introduced a thin and stretchable ion sensor array with an open cellular structure [46] (Figure 4B). A H^+^ sensing unit was prepared using the tridodecylamine ionophore and showed an excellent sensitivity and selectivity of the pH response. Moreover, the sensor had excellent stretch ability and fluidic permeability through a porous structure, as well as a rational physical design, which give it a potential application in integrated electronic devices for skin and internal organs. The successful implementation of the above cases shows that HIs can be used to manufacture various forms of wearable pH sensors.

In 2019, Niu’s group reported on an integrated wearable sensor chip that included an HIs–based pH sensor [79] (Figure 4C). The flexible chip could maintain potential stability under bending at 30, 60 and 90°. The flexible chip also showed high sensitivity (−56.0 ± 0.6 mV/pH) and excellent selectivity. In addition, the sensor chip was successfully applied to monitor the pH of human sweat, and the test results were consistent with those of commercial pH meters. Subsequently, Niu et al. further reported on a highly stretchable fiber–based ion–selective electrode (ISE) prepared by coating an ion–selective membrane (ISM) on a stretchable gold fiber electrode [80] (Figure 4D). The pH electrode was fabricated by using HIs of tridodecylamine. The prepared fiber sensors showed high stretchability up to 200% strain and the Nernst slope of the ion response exhibited a slight fluctuation from −59.2 to −57.4 mV/pH between 0 and 200% strain. The fiber sensors showed a low RSD of only 1.1%. The prepared fiber electrodes were further integrated into a hair band to monitor changes in biomarkers, including pH; the results displayed a high accuracy compared to ex situ analysis.

HIs show good compatibility in various wearable systems. However, some HIs, such as tridodecylamine and 4–nonadecylpyridine, exhibit biotoxicity upon skin contact [81,82]. Additionally, HIs are costly, which may be the reason why they are not commonly used in wearable pH sensors. As mentioned earlier, the rational design of flexible sensor chips and the optimization of the HIs fabrication process are key to the application of HIs in wearable devices.

### 3.3. Metal Oxides (MO_x_)

MO_x_ with micro–nano structures have been widely used in the fabrication of pH sensors due to their unique pH-sensitive properties and advantages, such as high mechanical strength, low cost, thermal stability and weather resistance [48,52,57,83,84,85,86,87,88,89,90,91,92,93,94,95,96,97,98,99,100,101]. MO_x_–based pH sensors can achieve a fast response in different environments and have the advantages of long life and easy miniaturization, which makes such sensors suitable for applications in wearable healthcare systems. Among various MO_x_, IrO_x_ has attracted more attention due to their advantages of a wide pH detection range, fast response, high precision and high durability. However, the high cost of the element Ir is a concern for manufacturing on a large scale. Additionally, the pH response mechanism of MO_x_–based pH sensors has not yet been clearly determined. Here, we will discuss the pH response mechanism of MO_x_–based pH sensors and review the research progress of MO_x_ –based wearable pH sensors in recent decades.

pH-response mechanism for MO_x_. The pH–sensitive mechanism for MO_x_ materials is quite complex and strongly correlates with their surface properties. As early as the 1980s, Fog and Buck demonstrated near–Nernstian pH responses for a series of MO_x_ (PtO_2_, IrO_2_, RuO_2_, etc.) in a wide pH range of 2–12. They proposed a few possible mechanisms^48^: (1) ion exchange through surface –OH groups; (2) H^+^-involved redox reaction with MO_x_; (3) H^+^–intercalated redox reaction; (4) H^+^–involved oxygen deficit redox reaction; and (5) electrode corrosion. The first mechanism (1) is similar to that of a traditional glass electrode, in which the surface –OH group plays the role of proton transfer conductor. The latter four mechanisms are proton-involved redox reactions. McMurray et al. showed that mechanism (4) was the closest explanation [102], while Trasatti, Mihell and Atkinson discussed the possibility that the pH response was caused by the first mechanism (1) of ion exchange [103,104].

**Figure 5:**
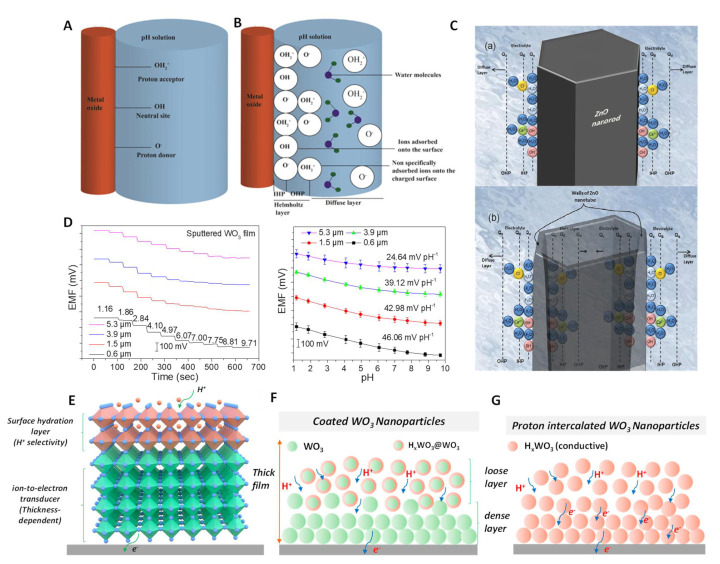
pH response mechanism for MO_x_. (**A**) Schematic representation of the site–binding theory to illustrate the response mechanism for MO_x_–based pH sensors. (**B**) The formation of the EDL structure on the surface of MO_x_. Reprinted with permission from [34], Copyright (2020) Elsevier. (**C**) Schematic illustration of the charge distribution at ZnO for (**a**) nanorod and (**b**) nanotube structures. Reprinted with permission from [98], Copyright (2009) MDPI. (**D**) The thickness dependence of pH sensitivity for WO_3_. (**E**) pH response model according to typical SC–ISEs, including the surface hydration layer (H^+^ recognition) and bulk ion–to–electron transducer layer. (**F**) Limited hydration for coated WO_3_ nanoparticles. (**G**) Complete hydration by the proton intercalation of WO_3_. Reprinted with permission from [109], Copyright (2022) John Wiley and Sons publications.

Recently, Dahiya and coworkers proposed the H^+^ response mechanism for MO_x_ based on a site–binding model [34]. It was suggested that surface oxygen–containing groups (–O–, –OH and –OH_2_^+^) could be formed spontaneously on the surface of MO_x_ in aqueous solution (Figure 5A). This layer was called the inner Helmholtz plane (IHP; Figure 5B). The charged sites could further absorb another charged layer, i.e., the outer Helmholtz plane (OHP). An electric–double layer (EDL) structure was formed at the MO_x_/solution interface [101,105,106,107]. Relatively free charged ions exist in the diffusion layer due to heat or electricity [108]. The EDL potential is sensitive to the pH changes in solution due to changes in the equilibrium state of the MO_x_ surface. The authors proposed an equation to illustrate the correlation between potential and pH according to the classic Gouy–Chapman–Stern EDL model [34].

The above mechanism is a good explanation for the higher sensitivity of materials with a larger specific surface area due to the presence of more surface sites. For example, compared with ZnO nanorods, the sensitivity of ZnO nanotubes is greatly improved (from −28.4 to −45.9 mV/pH) [98], as shown in Figure 5C. Although the sensitivity between different MO_x_ can be attributed to different surface sites caused by crystalline structures, it is difficult to prove this explanation using existing technical means. In addition, the relationship of sensitivity to the thickness dependence of MO_x_ is difficult to explain because the number of surface sites is independent of film thickness.

According to our recent study, the pH sensitivity of sputtered WO_3_ films has a strong correlation with thickness [109]. The sensitivity decreases with the thickness of WO_3_, as shown in Figure 5D. In addition, the response pH range either decreases with the thickness. To this end, we proposed ‘surface hydration layer’ mechanism to explain this phenomenon. The surface hydration layer (H_x_WO_3_) can be spontaneously formed upon WO_3_ contacting aqueous solution, which determines the pH selectivity (Figure 5E). The bulk WO_3_ layer functions the ion–to–electron transformation. The limited degree of hydration necessitates that proton and electrode must pass through a highly resistive diffusion pathway, which resulting in low sensitivity (Figure 5F). For WO_3_ with a higher degree of hydration or full hydration, all WO_3_ nanoparticles are transformed into conductive and selective H_x_WO_3_ (Figure 5G). As described in our report, compared with pristine WO_3_, the sensitivity of H_x_WO_3_ increased from −25.5 to −52.5 mV/pH. This response mechanism can be extended to other MO_x_. The response process of MO_x_ can be described as a reversible reaction: MO_x_ + y e^−^ + y H^+^ ⇋ H_y_MO_x_. This reversible reaction meets with thermodynamic equilibrium. Therefore, the potential for the process can be further described as a Nernst equation:

(3) $$E=E^{0}+\frac{RT}{yF}ln\frac{a\left(MO_{x}\right)a{\left(H^{+}\right)}^{y}}{a\left(H_{y}MO_{x}\right)}$$

where $E^{0}$ represents the standard electrode potential of the reaction; *R*, *T* and *F* have their usual meanings; $a\left(H^{+}\right)$ is the activity (or concentration in calibration) of H^+^ in the test aqueous solution; and $a\left(MO_{x}\right)$ and $a\left(H_{y}MO_{x}\right)$ represent the respective activities of MO_x_ and H_y_MO_x_, which are constant since they are solid species.

In fact, the mechanism of the surface hydration layer may be more reasonable than site–binding theory. First, surface hydration layer theory can explain the thickness dependence of sensitivity. Secondly, the spontaneous formation of the surface hydration layer can be determined by observing the valence state of metal ions using XPS. Finally, the theory can also explain the difference in the sensitivity of different specific surface areas due to the formation of hydration layers in different areas.

**Figure 6:**
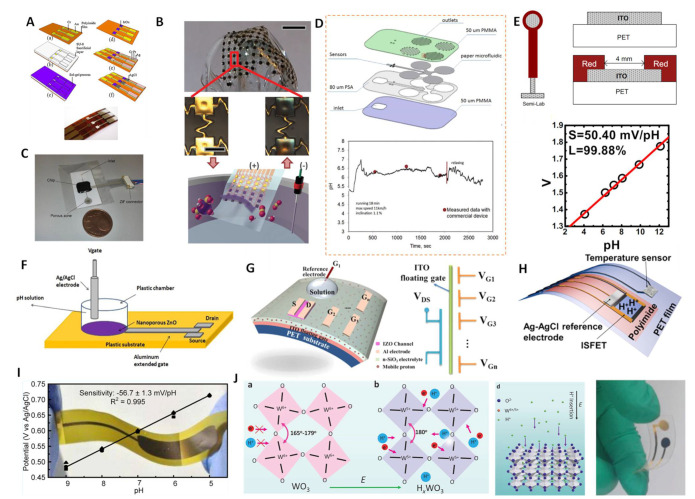
MO_x_–based wearable pH sensors. (**A**) The preparation process for IrO_x_–based flexible pH sensors. Reprinted with permission from [52], Copyright (2011) Elsevier. (**B**) Picture of thin compliant array of pH sensors. Reprinted with permission from [37], Copyright (2014) John Wiley and Sons publications. (**C**) Integrated silicon chip (with paper at the inlet for absorbing experiments). Reprinted with permission from [110], Copyright (2016) Elsevier. (**D**) Schematic representation of the fabrication steps of the micro-fluidic chip. The figure shows the real-time monitoring image of the human sweat pH value from the microfluidic chip. Reprinted with permission from [111], Copyright (2017) Elsevier. (**E**) Schematic and cross–section of the ITO/PET electrode. The figure shows the pH linear response curve. Reprinted with permission from [112], Copyright (2012) Elsevier. (**F**) Flexible pH sensor based on extended gate transistor. Reprinted with permission from [97], Copyright (2014) Elsevier. (**G**) Flexible pH sensor based on an IZO neuromorphic transistor with multiple gate electrodes. Reprinted with permission from [51], Copyright (2015) Spring Nature. (**H**) A wearable ion-sensitive filed effect transistor (ISFET) integrating flexible pH and temperature devices. Reprinted with permission from [113], Copyright (2017) American Chemistry Society. (**I**) Flexible WO_3_–based pH sensor on metal substrate with −56.7 ± 1.3 mV/pH sensitivity. Reprinted with permission from [93], Copyright (2014) American Chemistry Society. (**J**) Structures of WO_3_ before and after proton intercalation (H_x_WO_3_). The image on the right shows a schematic of the insertion of a proton into the lattice of WO_3_ and the photograph on the left shows the flexible electrode after proton intercalation. Reprinted with permission from [109], Copyright (2022) John Wiley and Sons publications.

MO_x_–based wearable pH sensors. IrO_x_ has become the most widely used wearable pH sensor due to its high sensitivity, good biocompatibility and fast response. In 2011, Huang et al. fabricated flexible IrO_x_–based pH electrodes using a sol–gel fabrication process [52]. The preparation is shown in Figure 6A. The flexible electrode showed reversible responses with a sensitivity of around −52 mV/pH in the pH range of 1.5–12. Meanwhile, the sensor exhibited good stability, repeatability and selectivity and a fast response. Subsequently, Rogers and coworkers proposed a low–modulus elastomer-based IrO_x_ pH sensing array [37] (Figure 6B). In vitro testing revealed a super–Nernstian sensitivity of −69.9 ± 2.2 mV/pH. This flexible sensing array enabled non–invasive spatiotemporal pH mapping on the surface of the beating heart due to its good flexibility and thirty-channel pH sensing array. This work demonstrated the use of an IrO_x_–based pH sensor for the pH distribution mapping of ischemic heart endocardium.

To solve this issue, Toonder et al. proposed a flexible and integrated microfluidic device based on a silicon chip [110] (Figure 6C). The filter integrated in the inlet part can absorb any liquid on the surface. The liquid then fills the microchannels and sensing cavity by capillary action. The integrated IrO_x_ sensor chips showed a pH–sensitivity up to −61 ± 1 mV/pH; however, the standard electrode potential between different electrodes had a large bias, which could be solved by calibrating each individual electrode. The special microfluidic design enabled the device to generate continuous and prolonged sensor signals, laying a good foundation for the further development of flexible wearable devices. Anastasova and coworkers further developed IrO_x_ thin film electrodes for human sweat pH detection [111]. Their patch contained paper microfluidic channels, embedded flexible microneedle sensors and a wireless potentiostat (Figure 6D). The flexible sensor exhibited a high sensitivity of up to −71.9 mV/pH and a low RSD of 1.1%. Real–time tests on human motion showed consistent results between the wearable patch and commercial sensors during maximum operation at a sustained speed (Figure 6D below). Dahiya et al. proposed a wearable pH sensor platform with an integrated IrO_x_ pH sensor and inductively coupled wireless transmission system [114]. The sensitivity of the sensing platforms was in the range of −65 to −75 mV/pH. In addition, the sensor maintained a normal potential response under bending at 30, 45 and 90°, and the test results in artificial sweat were consistent with commercial pH meters, indicating that the wireless pH sensing platform is suitable for wearable systems.

In addition to IrO_x_, some other metal oxide-based extended gate field effect transistors (EGFETs) and ion-sensitive field–effect transistors (ISFET) have also been used to fabricate wearable sensors. Lai et al. reported on a fabrication method for mass-producible flexible EGFET sensing electrodes [112]. Indium tin oxide (ITO) layers were deposited on PET substrates (ITO/PET) as a pH–sensitive membrane (Figure 6E). The ITO layer with lower sheet resistance had a higher sensitivity, with an average sensitivity of −50.1 mV/pH in the range of pH 2–12, and higher potential reproducibility (the standard deviation of E^0^ was ±1.7 mV/h). Meanwhile, the flexible sensing EGFET had a strong anti–light interference ability and could be stored for a long time (more than 55 days). Subsequently, Maiolo et al. demonstrated the fabrication of a fully flexible EGTFT pH sensor on a polymer substrate using nanoporous ZnO [97] (Figure 6F). The nanoporous ZnO–based pH sensor showed a near–ideal Nernst response of −59 mV/pH. Liu et al. further fabricated flexible indium zinc oxide (IZO) –based neuromorphic transistors on flexible substrates for pH sensing [51] (Figure 6G). When the device was operated in a quasi–static dual–gate synergic mode, it showed a considerable pH sensitivity of −105 mV/pH. This work provided a new strategy for fabricating biochemical sensors with a high sensitivity, fast response and ultralow power consumption. Takei and coworkers further developed a flexible sweat pH sensor based on an InGaZnO–based ISFET [113] (Figure 6H). The sensor could keep the potential stable under a bending curvature of 13 mm. For the first time, the ISFET-based sensing device was worn by a human, and changes in pH and temperature during human movement were successfully detected, which verified the feasibility of the concept. The sweat measurement results were consistent with in situ technical results.

WO_3_ has also been used in the fabrication of wearable pH sensors due to its excellent biocompatibility, chemical stability and low cost. Santons and coworkers reported on a WO_3_–based wearable pH sensor that included a flexible reference electrode [93]. The flexible sensor showed a high sensitivity of −56.7 ± 1.3 mV/pH (Figure 6I). However, the sensor had a large response time of 23–28 s, which was unfavorable for the timeliness of the data in the wearable device requirements. Very recently, we proposed a flexible wearable pH sensor based on lattice proton intercalation with WO_3_ [109] (Figure 6J). The intercalated H_x_WO_3_ had a high reversible response sensitivity of −52.5 and −51.1 mV/pH, excellent selectivity and resistance to light and gas. It should be noted that the proton intercalation of WO_3_ revealed an ultrafast response (<5 s). The integrated sensing chip was combined with a miniature potentiometer to successfully monitor the pH value on a mobile phone in real time through Bluetooth transmission. This work presented a general strategy for lattice proton intercalation, which provides a new avenue for further applications of MO_x_–based wearable pH sensors.

Among many metal oxides, IrO_x_ is the most widely used due to its high sensitivity and fast response; however, its cost is high. MO_x_–based EGFET and ISFET have not demonstrated a rapid response time, despite their high sensitivity. WO_3_–based potentiometric pH sensors have great potential due to their advantages of low cost, high stability and good biocompatibility. However, intrinsic WO_3_ usually exhibits a sub–Nernst response and a slower pH response. The proton intercalation methodology offers a strategy for improving the sensitivity [109]. The question of how to further enhance the comprehensive performance of MO_x_–based pH sensors is deserving of further attention.

### 3.4. Wearable pH Sensors Based on Other Materials

**Figure 7:**
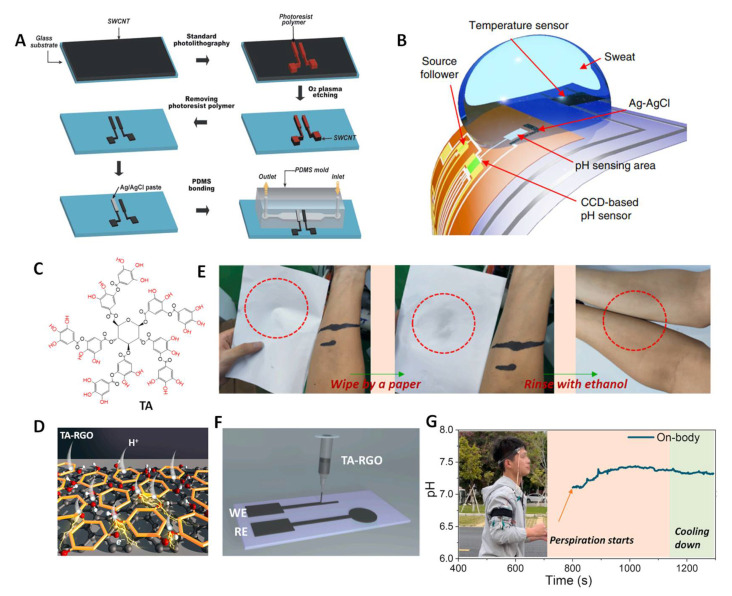
(**A**) The fabrication of a microfluidic pH-sensing chip. Reprinted with permission from [47], Copyright (2014) Royal Society of Chemistry. (**B**) A CCD–based flexible pH sensor integrated with a temperature sensor. Reprinted with permission from [115], Copyright (2018) Spring Nature. (**C**–**G**) Graphene–tannin supramolecule–based wearable potentiometric pH sensors. (**C**) Molecular structure of tannin (TA). (**D**) A schematic of TA–RGO supramolecular aggregates based on their π-π conjugation interaction. (**E**) Photographs of TA–RGO on the skin to test adhesion. (**F**) The wearable pH device was fabricated by directly printing TA–RGO on a flexible substrate. (**G**) Real–time sweat test of the flexible pH sensor. Reprinted with permission from [116], Copyright (2022) Elsevier.

In addition to PANI, HIs and MO_x_, some other materials have also been used to fabricate wearable pH sensors. Seong et al. developed a microfluidic pH sensing chip based on single-walled carbon nanotubes (SWCNTs) [47]. The specific preparation process is shown in Figure 7A. The SWCNTs thin film acted both as an electrode and a pH–sensitive membrane. The miniaturized SWCNT electrodes based on flexible PET substrates showed a Nernst slope and good selectivity. It should be noted that the SWCNT electrode showed high potential stability under the flowing state. Therefore, the sensor was suitable for the fluidic analysis of, for example, cell metabolism. Takei and coworkers developed a flexible charge–coupled device (CCD) –based super–Nernst pH sensor [115] (Figure 7B). The CCD–based pH sensor could cycle through the accumulation of electron charge transfer, reaching a sensitivity of about 240 mV per pH unit, which is about four times the theoretical limit of the Nernst response. In addition, an integrated flexible temperature sensor could be used to simultaneously compensate for temperature-dependent pH and detect skin temperature, enabling the real-time monitoring of human sweat pH and epidermal temperature. This CCD–based flexible pH sensor can be extended to the detection of other ions, providing a reference for the development of highly sensitive flexible detectors for the detection of other chemicals. Very recently, our work on tannin–graphene (TA–RGO) supramolecular aggregates suggested that they could be used as H^+^–sensitive materials to fabricate wearable pH sensors [116] (Figure 7C,D). The abundant phenolic hydroxyl groups in TA provide the membrane solution with strong adhesion, such that the material can be strongly attached to the electrode substrate without the use of a binder, thereby improving the potential stability and biocompatibility (Figure 7E). The phenolic hydroxyl group in TA is also a pH–responsive site, and the response mechanism is a classical proton–coupled electron transfer process. RGO induces the formation of TA supramolecules and enables ion–electron transduction based on π–π conjugation. The fabricated sensor exhibited a good reversible pH response with a slope of −52.4 ± 0.7 mV/pH and a low RSD of 1.3%, as well as excellent selectivity and anti–interference performances. A wearable pH device was fabricated by directly printing TA–RGO on a flexible substrate using a dispensing printing technique (Figure 7F), and it successfully detected pH changes during human exercise (Figure 7G). This work offered a concept of self–adhering wearable electrochemical pH sensors.

In addition to potentiometric pH sensors, a few optical pH sensors have also been successfully used in wearable devices. Rosace and coworkers proposed a wearable optical pH sensor [117]. It was fabricated based on a cotton fabric by a treatment with pH-sensitive organically modified silicate (ORMOSIL). The sensor was fitted with miniaturized and low–power wireless electronics, which displayed high accuracy and could work effectively in the pH range of 3–11 with a resolution of 0.2. In addition, the sensor was successfully applied to the pH monitoring of sweat during exercise in humans. Very recently, Kong et al. reported on a colorimetric patch assay for the pH monitoring of sweat [118]. The patch was fabricated using a superhydrophilic thermoplastic polyurethane nanofiber modified with silica nanoparticles on a superhydrophobic substrate. This wettability contrast could efficiently collect the sweat. The colorimetric patch exhibited a pH detection range of 4.4–7.4 with colors that could be distinguished by the naked eye. The practical suitability of the sensing patch was demonstrated by the quantitative analysis of sweat composition after physical exercises. The success of the above cases fully demonstrates the application potential of silicon–based wearable pH sensors in health and personalized medicine.

The successful application of the above materials has expanded the types of pH–sensitive materials and accelerated the development of wearable pH sensors. In future, a comprehensive evaluation of the analytical performances of these sensors using factors such as response time, reversibility, flexibility and stability are necessary.

To clearly compare the differences between various pH–sensitive materials and applications, the sensing properties of PANI, HIs and MO_x_ are shown in Table 1. It can be seen that most of the pH–sensitive materials could offer Nernstian sensitivity and even a super–Nernstian response through assay integration, optical readout and electronic techniques. In addition, their pH measurement range covers the pH of sweat (4–8). However, their response times have a broad range. From the viewpoint of practical applications, a rapid response (~5 s) should be encouraged. Overall, the basic pH analytical performances of current pH sensors should satisfy the requirements for wearable application. A standardized test protocol for on–body evaluation is much more urgent.

## 4. Outlook

In this review, we mainly discussed the development of several commonly used materials for wearable pH sensors, including PANI, HIs, MO_x_ and other materials. The pH–sensitive mechanism of the above materials was analyzed, and the recent achievements of these materials in the fabrication of flexible and wearable sensors were reviewed.

The pH value of human biological tissue fluids is an important physiological indicator, reflecting a large amount of biological information related to health. With the development of miniaturized integration technology, wearable pH sensors have expanded from in vitro detection to in vivo detection and even cell analysis. This puts forward higher requirements for pH sensors, such as sensitive detection in a small pH range, a faster response and good biocompatibility. Additionally, the potential reproducibility of the sensor is also very important, as it determines whether different electrodes can be directly interchanged without the calibration of the actual test. PANI and HIs show satisfactory sensitivity and selectivity. However, biocompatibility may be an issue requiring further consideration. For example, PANI alone is less cytotoxic, but its low-molecular-weight byproducts could have significant carcinogenic effects, such as those of benzidine [76]. HIs, such as tridodecylamine and 4-nonadecylpyridine, exhibit biotoxicity upon contact with skin [81,82].

MO_x_ are a classic type of pH–sensitive materials that exhibit good biocompatibility. For example, the most widely used IrO_x_ not only shows a low drift value, Nernst sensitivity and fast response performance, but also good biocompatibility [119]. Meanwhile, MO_x_ also have the advantages of chemical stability, high mechanical strength, weather resistance and a rapid response in complex environments [34,48,120]. In addition, the performance of MO_x_ can be further improved by intercalation, nanostructures and composites, which make it possible to build a low–cost, high-performance wearable pH sensor [121,122,123]. It is worth noting that the pH response mechanism of MO_x_ needs to be further verified to provide theoretical support for MO_x_–based wearable pH sensors. Overall, MO_x_–based wearable pH sensors have promising prospects and a predictable future in wearable systems.

In addition to the advancement of sensing materials, the improvement of wearable devices is also a key direction of future development. For example, efficient microfluidic sweat collection systems, a low–energy consumption of data acquisition, processing and transmission modules and more robust packaging are among the key factors driving wearable pH sensors beyond their initial stages. Additionally, a reasonable standard measurement protocol and the development of a more stable solid–state reference electrode should be introduced as soon as possible in the future—this is key to obtaining valid data. Finally, the relevance of the test results of human biofluids to biomedicine should also receive more attention.

## Figures and Tables

**Table 1:** Comparison of analytical characteristics of wearable PANI, HIs and MO_x_ pH sensors and sensors based on other materials.

Materials	pH Range	Sensitivity (mV/pH)	RSD (%)	Response Time	On-Body Test	Ref.
PANI	3–7	−57.5 ± 3.3	5.7	10–25 s	50 min	[54]
PANI	4–8	−60.6 ± 1.8	3.0	–	–	[70]
PANI	2–12	−58.2	–	<10 s	–	[71]
PANI	3–8	−63.3 ± 1.5	2.3	–	30 min	[72]
PANI	4–8	−54.4 ± 1.9	3.5	<1 s	30 min	[22]
PANI	4–8	−56.2	–	–	30 min	[73]
PANI	4–8	−58.5 ± 0.7	1.2	<20 s	–	[74]
PANI	4–10	−50	–	~12 s	–	[69]
PANI	3–8	−59.6	–	<30 s	Chicken skin	[75]
HIs (I)	3–11	−59.2 ± 3.0	<5	5 s	–	[28]
HIs (I)	6.4–7.4	−51.8	–	–	–	[46]
HIs (I)	4–7.5	−56.0 ± 0.6	1.0	–	1 h	[79]
HIs (I)	4–8	−58.2 ± 0.6	1.1	–	30 min	[80]
IrO_x_	1.5–12	−51.1 to –51.7	–	2 s	–	[52]
IrO_x_	4–9	−69.9 ± 2.2	3.1	0.5 s	Rabbit and donated human hearts	[37]
IrO_x_	2–10	−61 ± 1	1.6	–	–	[110]
IrO_x_	–	−71.9 ± 0.8	1.1	–	>40 min	[111]
IrO_x_	4–9 or 2–10	−65 to −75	–	<2 s	–	[114]
ITO	2–12	−50.1 ± 4.1	8.2	–	–	[112]
ZnO	1–9	−59	–	–	–	[97]
IZO	4–10	−105	–	–	–	[51]
InGaZnO	3.3–11	−51.2	–	–	~20 min	[113]
WO_3_	5–9	−56.7 ± 1.3	2.3	23–28 s	–	[93]
H_x_WO_3_	2–8	−53.6 ± 1.6	3.0	<10.5 s	~20 min	[109]
SWCNTs	3–11	−59.7 ± 1.5	2.5	–	–	[47]
CCD	2.8–11.2	−240	–	–	100 s	[115]
TA-RGO	1–10	−52.4 ± 0.7	1.3	–	20 min	[116]

## Data Availability

Not applicable.
